# Cloning and expressing of interleukine 2 in amniotic membrane-derived mesenchymal stem cells, as a potent feeder layer

**DOI:** 10.22099/mbrc.2021.38845.1566

**Published:** 2021-06

**Authors:** Saeid Anvari, Farshad Foroughi, Mehdi Azad, Amirhosein Maali, SafarAli Alizadeh, Mohammad Hossein Ahmadi

**Affiliations:** 1 Department of Medical Biotechnology, Faculty of Allied Medicine, Qazvin University of Medical Sciences, Qazvin, Iran; 2 Student Research Committee, Qazvin University of Medical Sciences, Qazvin, Iran; 3 Department of Immunology, School of Medicine, Qazvin University of Medical Sciences; 4 Department of Medical Laboratory Sciences, Faculty of Allied Medicine, Qazvin University of Medical Sciences, Qazvin, Iran; 5 Student Research Committee, Pasteur Institute of Iran, Tehran, Iran; 6 Medical Microbiology Research Center, Qazvin University of Medical Sciences, Qazvin, Iran

**Keywords:** Interleukin-2, Mesenchymal stem cells, Plasmids, Transfection

## Abstract

The application of mesenchymal stem cells (MSCs) is rapidly expanding due to their unique properties in cell therapy, especially as the feeder layer in the* ex-vivo *expansion of immune cells. Also, Interleukin 2 (IL-2) is an essential human cytokine in the expansion of hematopoietic precursors and progenitors, i.e., NK cells and T cells, while there is no endogenous expression of IL-2 in MSCs. This study aimed to examine the potency of amniotic membrane (AM)-MSCs as the IL-2 secretory cells. *IL-2-*containing pCMV3-C-GFPspark shuttle vector was transformed in *E.coli DH5-alpha. *After cloning, the plasmid DNA was extracted and transfected in isolated AM-MSCs, by lipofectamine-2000. Then, the RNA and protein expression levels of exogenous IL-2 were evaluated 3 to 15 days after transfection, using ELISA and qRT-PCR. Fluorescent microscopy and flowcytometry assays were used for evaluating the GFP-positivity of transfected AM-MSCs, as IL-2 expression control. There was a significant increase in RNA expression of exogenous IL-2 in transfected AM-MSCs in 3 to 15 days after transfection. (p<0.001) Also, IL-2 concentration released in the medium was increased in 3rd day after transfection (611 pg/ml). However, the RNA and protein expression of IL-2 was reduced through passing the time. The results show AM-MSC is a suitable host for the expression and secretion of IL-2 as a critical cytokine in the *ex-vivo *expansion of hematopoietic precursors and progenitors, i.e., NK cells and T cells. Also, the survival time of IL-2 expression in AM-MSCs was long enough for use as a feeder layer.

## INTRODUCTION

Cell therapy is a novel approach to cellular replacement or amplification aimed at tissue regeneration or restores lost functions [1]. Cellular immunotherapy is a high-tech medication which amplifies the cell-mediated immunity in *ex-vivo/in-vitro* condition applicable in various diseases, e.g., cancers. Nowadays, the most specialized treatment for malignancies is cell therapy [2]. The most efficient cells used for this aim are cytotoxic lymphocytes, such as cytotoxic T cells (acquired immunity), and natural killer (NK) cells (innate immunity). NK cells are cellular immune components with a lymphoid appearance that can detect and kill abnormal cells without prior exposure to the antigen and without the need for activation and antigen presentation. Inactive NK cells circulate in the peripheral blood, but after activation with cytokines, they can undergo diapedesis into the pathogen-infected sites and malignant cells [3, 4]. As a result, they are an ideal choice for altering and manipulating, and ultimately applying in immune cell-mediated cancer therapy. In addition, NK cells are involved in suppressing the graft versus host disease (GvHD), accelerating transplant acceptance, and responding to graft versus leukemia (GvL) effect during allogeneic hematopoietic stem cell transplantation [5, 6]. During the GvL effect, NK cells increase the efficiency of the transplantation by eradicating the remaining leukemic cells. Therefore, transplantation of bone marrow donor NK cells can be considered as part of the post-transplant treatment regimen [7-9]. Also, the efficacy and safety of NK cell transplantation in patients with metastatic melanoma, renal cell carcinoma, resistant Hodgkin lymphoma, and acute myeloid leukemia have been identified. Today, efforts are being made to use NK cell therapy to treat solid tumors such as ovaries and breast cancer [10-12].

Despite all efforts in NK cell therapy in cancer, the low number of NK cells in the peripheral blood circulation is a major challenge in using these cells in immunotherapy. Restrictions on stimulating the proliferation of NK cells in *in-vivo* conditions due to host rejection, competition with host lymphocytes, or suppression by host T-regulatory cells (T-reg) or myeloid suppressor cells force researchers to identify methods for *ex-vivo *expansion of NK cells [13]. In this regard, NK cell precursors derived from bone marrow, umbilical cord, or peripheral blood are cultured in the presence of cytokines and feeder cells to the expansion of the ideal number of NK cells for clinical application [14]. Various studies have shown that mesenchymal stem cells (MSCs), as feeder layer, play an important role in the reproduction and differentiation of NK precursors into mature and efficient cells through physical contact and the secretion of growth mediators and various cytokine [15]. Interleukin (IL)-2 is a critical human cytokine to the proliferation and expansion of NK cells. However, MSCs are unable to secrete IL-2 [16]. In this study, we aim to generate an IL-2-expressing amniotic membrane-derived MSCs (AM-MSCs) to introducing a potent feeder cell in the proliferation of NK cell precursors.

## MATERIALS AND METHODS

**Isolation and primary culture of AM-MSCs** **: **In order to isolate MSCs from amniotic membrane tissue, the placenta was immediately placed in sterile saline and transferred to the cell culture laboratory of Qazvin University of Medical Sciences for further testing. After a selective cesarean delivery, the placenta was obtained regarding ethics approved by the ethics committee of Qazvin University of Medical Sciences (IR.QUMS.REC.1396.448). The amniotic membrane was separated from the placenta and washed three times by 1x PBS. The amniotic membrane was cut to 1cm × 1cm pieces and incubated with 1ml of 10x collagenase type IV (Gibco, USA) and 9ml of 1x PBS in 37°C for 4 hours. After incubation, the cellular suspension was filtered and washed twice by 1x PBS. After a mild centrifuge, the supernatant was removed and the pelleted cells were suspended in low-glucose Dulbecco’s modified eagle medium (DMEM-LG; Gibco, USA) supplemented by 10% of fetal bovine serum (FBS; Gibco, USA) containing 100 U/ml Pen-strep (Sigma-Aldrich, USA), in a T-75 flask. The medium was replaced per 72 hours. 

**Characterization of isolated AM-MSCs** **:** The flowcytometry assay was applied for the characterization of MSCs derived from the individual. In passage no.3, the AM-MSCs were immunophenotyped for the presence of CD90, CD105, CD44, CD166, and absence of CD45, CD34, and CD14. For this aim, the cells were washed with 1x PBS. After the cells were cultivated using 100µl of trypsin from the bottom of the flask, 1 ml of the complete medium was added to neutralizing trypsin. The suspension was centrifuged for 1 minute at 2000 RPM. After removing the supernatant, 250μl of 2% paraformaldehyde was added to the pellet. After incubation for 20 min at 4°C, the suspension was centrifuged for 1 min at 2000 RPM. Cellular sediment was washed three times with 1x PBS and suspended in 200 ml of 1x PBS. Anti-body conjugations and treatments were conducted by CD-marker detection kit (IQ products, Netherland), as manufacturer protocol. The reactions were read by flowcytometer (BD FACSCalibur™). 

**Plasmid transfection and cloning** **:** cDNA of IL-2 was obtained from NCBI, and after amplification, it was inserted in pCMV3-C-GFPspark shuttle vector (Sinobiological, cat no: HG11848-ACG) using KpnI and NotI restriction enzyme. E. coli (DH5-alpha strain) was applied for cloning. For this aim, E. coli had been cultured overnight in LB broth. After achievement to 0.5 OD (in λmax = 600nm), it was centrifuged at 4°C for 15 minutes at 5000 RPM. Then, 1.5 ml of 100mM CaCl_2_ was added to the pellet for sensitizing. The suspension was placed on ice for 30 minutes. After the incubation period, it was centrifuged at 4°C for 15 minutes at 5000 RPM. Then, 400µl of 100mM CaCl_2_ was added to the pellet. A total amount of 2µl of plasmid was added on suspension and placed on ice for 30 minutes. The thermal shock was done for 42 seconds on 42°C and 2 minutes on ice, respectively. The transfected suspension was incubated on LB broth at 37°C. The transfected colonies, which grew on kanamycin-contained LB agar (as differential culture medium) was cultured on LB broth.

**Plasmid extraction** **:** After an overnight culture of transfected hosts in LB broth, 15 ml of suspension was centrifuged at 4500 rpm for 5 minutes. Plasmid extraction was performed by plasmid extraction kit (RNA Biotech, Iran), as manufacturer protocol. The extracted plasmid was qualified by nanodrop and electrophoresis on 1.5% agar gel. 

**pDNA lipofection of AM-MSCs** **:** A total number of 6×10^4 ^cells/well of AM-MSCs were seeded on a 24-well plate to achievement to 90% confluent at transfection day. The lipofectamine-2000 (LF2000) transfection was optimized on 3 (LF2000):1 (pDNA) ratio, diluted in Opti-MEM (Gibco, USA), as manufacturer protocol. A total amount of 50µl of diluted LF2000/pDNA (containing 500ng of pDNA/well) was treated on adherent AM-MSCs. After five hours of passing from transfection, the medium was replaced with 500µl of DMEM-LG supplemented by 10% FBS and 100 U/ml Pen-strep. The transfected AM-MSCs were cultured for further tests. 

**The RNA expression level of exogenous IL-2 in transfected AM-MSCs** **:** The RNA expression level of exogenous IL-2 was performed in 3, 6, 9, 12, and 15 days after transfection. On test days, the total RNAs were extracted by Iraizol kit (RNA Biotech, Iran), as manufacturer protocol. The cDNAs were synthesized by cDNA kit (RNA Biotech, Iran) as manufacturer protocol. The SYBR-green quantitative real-time PCR (qRT-PCR) was used for the quantification of the RNA expression level of exogenous IL-2. Primer sequences were designed for IL-2-linked green fluorescent protein (GFP). The reactions were performed as follow: 1µl of each primer (10 pM), 10µl of 2xRB SYBR qPCR MIX (RNA biotech, Iran), 1µl of cDNA, and 7µl of dd-H_2_O, at the total volume of 20µl. The primer sequences of GFP were 5’-GCA AGC TGA CCC TGA AGT TC-3’ (forward) and 5’-GTC TTG TAG TTG CCG TCG TC-3’ (reverse). The primer sequences of internal control (GAPDH) were 5′-CAA TGA CCC CTT CAT TGA CC-3′ (forward) and 5′-TGG AAG ATG GTG ATG GGA TT-3’ (reverse). The strips were incubated at 95°C for 5 min, as initial denaturation. Then, 40 thermal cycles were conducted as bellow: 95°C for 20 sec (denaturation), 58°C for 20 sec (annealing), and 72°C for 30 sec (extension). The data were analyzed by REST software. 

**Evaluation of secreted IL-2 by ELISA** **:** Enzyme-linked immunosorbent assay (ELISA) was used to evaluate the medium level of IL-2 in 3, 6, 9, 12, and 15 days after transfection by human IL-2 ELISA kit pre-coated plates (BioLegend, USA), as manufacturer protocol. 

**Fluorescence microscopy and flowcytometry** **:** Observational qualification of GFP expression was evaluated by invert fluorescence microscopy at 3, 6, 9, 12, and 15 days after transfection. For this aim, transfected and untransfected AM-MSCs were fixed by 2% paraformaldehyde on a slide and observed by fluorescence microscope. The fixations applied as described in section 2.2. Also, the percent of GFP-positive AM-MSCs was estimated by flowcytometry of fixed cells in 2% paraformaldehyde.

**Statistical analysis** **:** Kruskal–Wallis test was used for evaluating the significance of qRT-PCR and ELISA results on different days. All statistical analyses were done by SPSS software. The significance level was considered 0.05. 

## RESULTS

**Figure 1:**
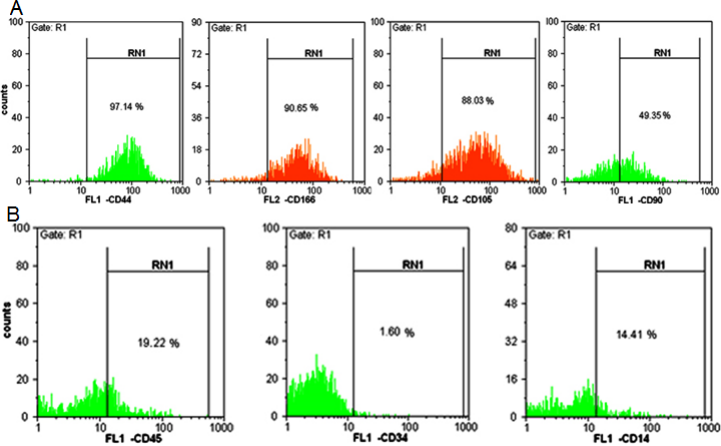
**Flowcytometric analysis of isolated AM-MSCs in the 3** ^rd^ **passage.** **A)** It shows the percentage of the target cell population with the surface CD marker expressing CD44, CD166, CD105, CD90 (positive markers). **B)** it shows the percentage of the target cell population with the surface CD marker expressing CD45, CD34, and CD14 (negative markers)

The isolated MSCs were characterized immunophenotypically by flowcytometry in terms of the presence of CD90, CD105, CD44, and CD166 and absence of CD45, CD34, and CD14. In the third passage, 97.14% of cultured cells were expressed CD44. Also, CD166, CD105, and CD90 were expressed on 90.65%, 88.03%, and 49.35% of cells, respectively. Also, 19.22%, 1.6% and 14.41% of cells did not expressed CD45, CD34 and CD14, respectively (Fig. 1).

**Figure 2:**
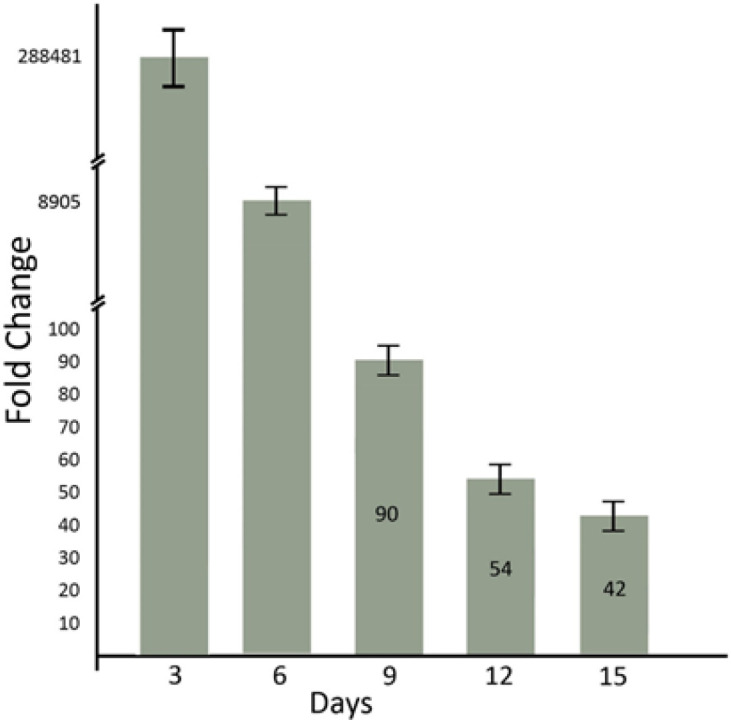
**The plasmid expression level in 3, 6, 9, 12, and 15 days after transfection. **The mRNA expression level of GFP was considered equivalent to exogenous IL-2 expression. There was no expression of plasmid mRNA before transfection. Also, GADPH was used as an internal control gene

For evaluating the expression level of exogenous IL-2 in transfected MSCs, the expression level of GFP (which was linked to IL-2 sequence in pDNA structure) was conducted as an equation for exogenous IL-2 expression level. In 3, 6, 9, 12, and 15 days after transfection of AM-MSCs, the relative fold-changes of the expression level of GFP were 284881 ± 15189, 8905 ± 485, 90 ± 12, 54 ± 8, and 42 ± 8, respectively. There was a significant difference in the expression level of exogenous IL-2 in transfected cells compared to control cells (untransfected) (p<0.001). Also, the expression levels of exogenous IL-2 were reduced through passing time (p<0.001) (Fig. 2).

**Figure 3:**
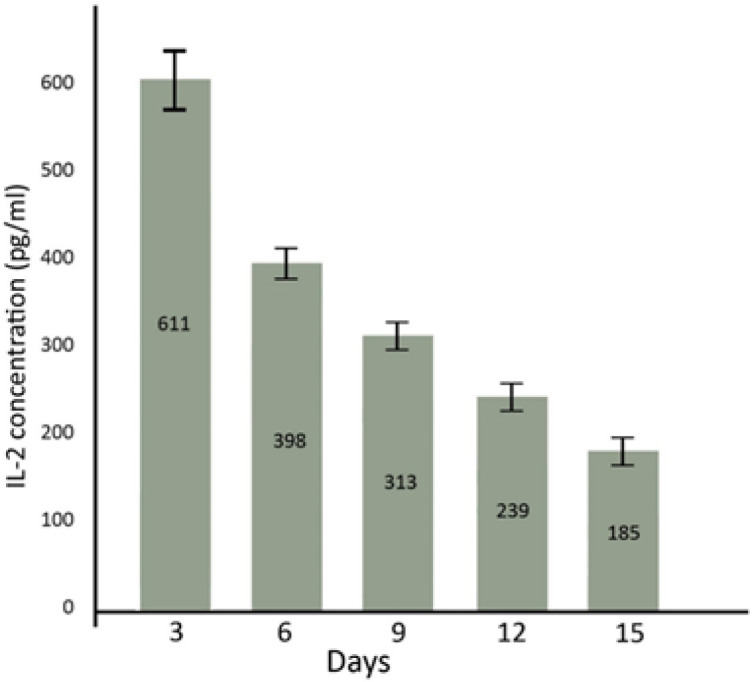
**IL-2 concentration** **in 3, 6, 9, 12, and 15 days after transfection. **There was a significant difference in IL-2 concentration of medium between transfected AM-MSCs and control (p<0.001).

ELISA results show that in 0 (control), 3, 6, 9, 12 and 15 days after transfection of AM-MSCs, the released IL-2 concentrations were 20 ± 0.01 pg/ml, 611 ± 35 pg/ml (p<0.01 compared to control), 398 ± 16 pg/ml (p<0.05 compared to control), 313 ± 13 pg/ml (p<0.01 compared to control), 239 ± 15 pg/ml (p<0.05 compared to control), and 185 ± 17 pg/ml (p<0.05 compared to control), respectively (Fig. 3).

**Figure 4:**
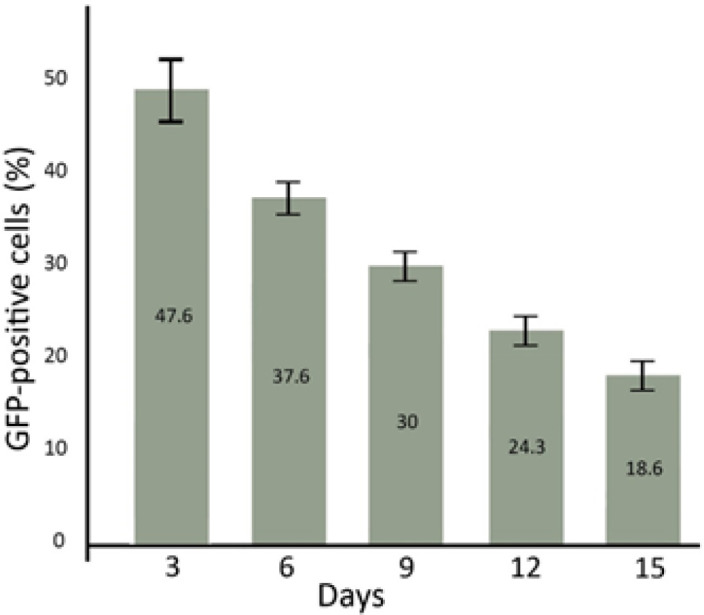
**The percentage of GFP-positive AM-MSCs in 3, 6, 9, 12, and 15 days after transfection. **The flowcytometric analysis showed that GFP expression is reduced through passing time

**Figure 5:**
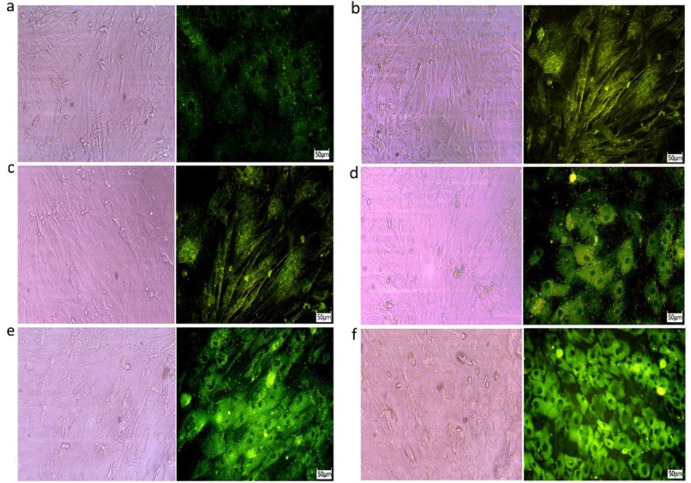
**The fluorescent microscopy of GFP-positive AM-MSCs in 3, 6, 9, 12, and 15 days after transfection. **Captures of invert light and fluorescent microscope in (a) untransfected AM-MSCs, (b) 3 days after transfecting pDNA in AM-MSCs, (c) 6 days after transfecting pDNA in AM-MSCs, (d) 9 days after transfecting pDNA in AM-MSCs, (e) 12 days after transfecting pDNA in AM-MSCs, and (f) 15 days after transfecting pDNA in AM-MSCs

As flowcytometry results, the percent of GFP-positive AM-MSCs was 47.6 ± 5.4, 37.6 ± 3.1%, 30 ± 2.5%, 24.3 ± 2%, and 18.6 ± 1.5% in 3, 6, 9, 12, and 15 days after transfection, respectively (Fig. 4). Also, fluorescent microscopy results show that GFP emission is reduced through the time passing (Fig. 5).

## DISCUSSION

The use of mesenchymal stem cells (MSCs) is rapidly expanding due to the unique properties of these cells in cell therapy as well as biological studies. The use of amniotic membrane as a rich source of MSC has received particular attention [17, 18]. Ethical considerations in isolating AM-MSCs from this tissue are minimal due to the nature of the biological biodegradability of the tissue [19]. As MSCs secrete various factors and cytokines needed for other cells to grow, they have been considered feeder cell layers in co-cultures, especially in immune system cells [15]. A study by Roubelakis et al. showed that AM-MSCs not only have the same characteristics as adult mesenchymal cells but also have some of the characteristics of embryonic stem cells, such as the expression of pluripotency markers, high proliferation rate in in-vitro condition and differentiate into various types of the cells. These make these cells as suitable candidates for cell therapy as well as gene transfer in in-vitro conditions [20]. MSCs are potentially a good candidate for the expansion of NK cells in cell culture media due to the innate secretion of IL-15 [21]. On the other hand, since these cells do not express IL-2, while it is essential for NK cell expansion, the exogenous IL-2 should be used [22, 23]. In this study, we tried to examine the ability of AM-MSCs as a secretory cell of IL-2 by transferring the IL-2 gene with the expression plasmid to the AM-MSCs. 

Since the transfer by cationic lipid methods such as lipofectamine compared to viral methods does not have intracellular gene control mechanisms, it is necessary to optimize the appropriate concentration of solutions for maximum gene transfer [24, 25]. According to the provider company’s recommendations, 2, 3, 4, and 5μl of lipofectamine and 5μg of pDNA were used for tests in the present study. Examining the amount of IL-2 secreted in all lipofectamine:pDNA ratios did not show a significant difference. No significant relationship was observed between concentrations of lipofectamine and pDNA [26]. It was shown that although a higher concentration of DNA was transferred to the cell at high concentrations of lipofectamine, it had no significant relationship with the expression of the transferred plasmid [26]. Researchers attribute this to the accumulation of DNA in the space around the nucleus, as well as the saturation of the plasmid expression in the cell. In fact, studies show that for each group of cells, a certain amount of plasmid and lipofectamine is effective in transfusion efficiency, and higher values have no effect on transgenic plasmid expression, even if plasmid delivery to the nucleus [27, 28]. Studies on melanoma mice and human lung carcinoma cells show that the amount of DNA transferred through the lipofectamine2000 in the nucleus forms a clamp that is virtually out of reach of the transcription system and cell translation, and this confirms that Transferring high-value DNA does not necessarily mean more efficient transcription [27]. Although the use of cationic lipids is less effective than electroporation and viral methods, our research on the transfection of AM-MSCs with lipofectamine2000 shows a 45% success rate of the process, which is very acceptable. A study on transfection using the cationic compounds showed that lipofectamine2000 was most effective in increasing transfection rate in MSCs [29]. Therefore, it seems that the use of cationic lipids not only does not have the challenges of using viral vectors but also has the least toxicity for the host cell (about 20%) [29, 30]. 

In our experiment, the expression level of IL-2 is reducing through passing time. Regarding with clinical application of this experiment, the *ex-vivo* culture of NK cells takes less than 10 days [31]. So, the high expression level of IL-2 in MSCs should be stable for at least 10 days. Our results showed that there is a high level of expression of IL-2 on the 15^th ^day after lipofection. It seems that IL-2 expression is suitable enough for ex-vivo expansion of NK cells aimed at immunotherapy. 

Finally, it is suggested that MSCs obtained from different origins, especially bone marrow, can be subjected to the same experience for comparison with AM-MSCs in terms of IL-2 expression potency. Also, it is possible to use permanent genome transfer methods, such as viral methods, for the expression of the *IL-2* gene as a substitute for the exogenous IL-2. Another suggestion is that NK progenitor cells can be cultured on IL-2-expressing AM-MSCs. Also, the results of *ex-vivo *NK cell expansion on IL-2 expressing AM-MSCs should be compared to non-IL-2-expressing AM-MSCs.

## Supplementary Materials

Supplement Fig. 1
