# Supplementary material for: Cloning and expressing of interleukine 2 in amniotic membrane-derived mesenchymal stem cells, as a potent feeder layer
Source: Mol Biol Res Commun. 2021 Jun;10(2):63–71. doi: 10.22099/mbrc.2021.38845.1566 (PMC8310657; doi:10.22099/mbrc.2021.38845.1566)
Supplement: Supplement Fig. 1 [file mbrc-10-63-s001.pdf]

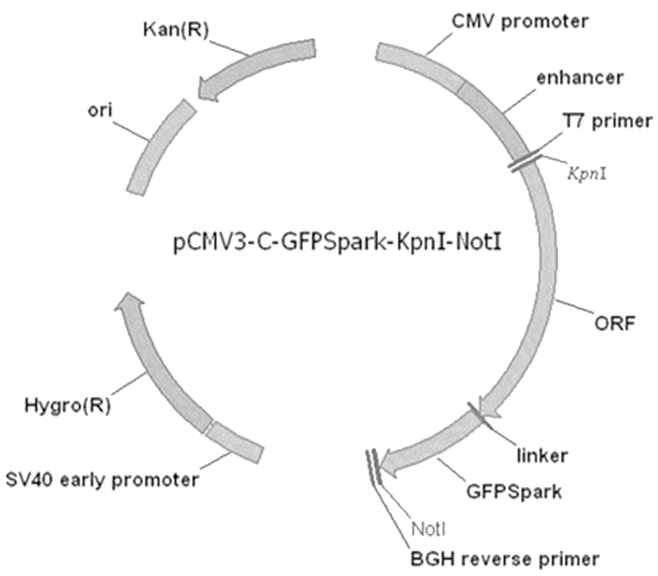

figure S1: Structure of pCMV-IL2-GFPspark vector used for IL-2 cDNA insertion. *KpnI* and *NotI* were used for restricting and cutting sites.

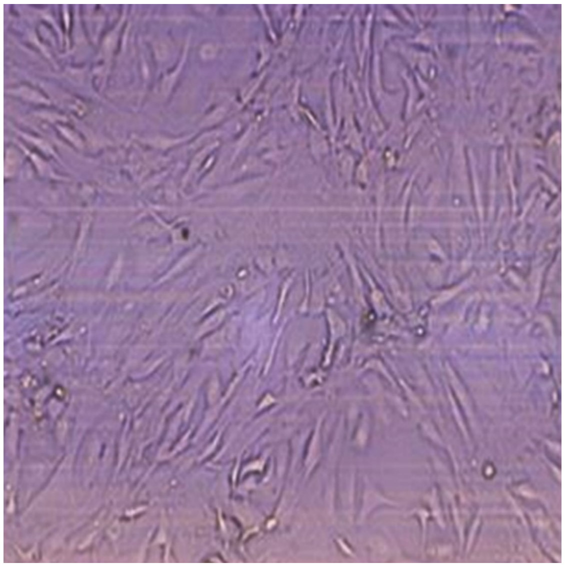

figure S2: Isolated AM-MSCs. AM-MSCs adhered to flask surface in 90% of confluency.
